# Effects of bed cleanliness on tucked and extended sleep-related lying postures of Japanese Black fattening cattle

**DOI:** 10.5713/ab.23.0137

**Published:** 2023-08-16

**Authors:** Shen Dan, Hidetoshi Kakihara, Michiru Fukasawa

**Affiliations:** 1 Graduate School of Agricultural Science, Tohoku University, Osaki, Miyagi 989-6711, Japan; 2 Western Region Agricultural Research Center, National Agriculture and Food Research Organization, Oda, Shimane 694-0013, Japan

**Keywords:** Bed Cleanliness, Japanese Black Fattening Cattle, Lying, Sleep-related Lying Posture

## Abstract

**Objective:**

Resting comfort may influence sleep-related lying postures in cattle. This study aimed to investigate the effects of bed cleanliness on tucked (TSP) and extended (ESP) head positions in sleep-related lying postures.

**Methods:**

The study was conducted over two experimental periods. In each period, four Japanese Black fattening cattle were assigned to the cleaning treatment (CL), in which bedding material was replaced once during each experimental period. Four cattle were assigned to the control treatment (CON) with no bed cleaning. Daily duration, bout frequency, and bout length of sleep-related lying postures were measured, and bed moisture, ammonia concentration in the air, plasma cortisol, and serotonin concentration were also measured within one week before and after cleaning treatment in each period.

**Results:**

The bed moisture and ammonia concentrations decreased in CL after bed cleaning. Following bed cleaning, the duration and bout frequency of TSP in CL decreased compared to that observed in CON, whereas ESP in CL increased after bed cleaning. Total duration of sleep-related lying postures and cortisol and serotonin levels did not differ between CL and CON.

**Conclusion:**

These results suggest that cattle in sleep-related lying postures shifted from tucked head positions to extended head positions in response to improved bed cleanliness.

## INTRODUCTION

Sufficient and good-quality rest plays an important role for cattle well-being. Comfort during resting is considered an on-farm welfare criterion [1]. Cattle usually spend most of their resting time in a lying position [2]. The effects of housing and management on lying postures of cattle have been previously studied. Calves spend a longer duration lying with their heads touching the flank or ground under housing that has environmentally enriched conditions [3]. Cows kept in deep-bedded free-stall barns spend more time lying with their head back or on the ground compared to those in tie stalls with concrete flooring [4]. Cows spend less time with their head touching the ground or their flank when the ground is cold and muddy [5]. Cows also spend less-than baseline time in lying postures with their heads against the frank after moving into an unfamiliar tie-stall [6].

Recently, some lying postures were suggested to be related to sleep. Sleep is characterized by a temporary period of inactivity and specific postures [7]. Ruckebusch et al [8] stated that cattle could only sleep when they are well accustomed to the surroundings. When in insecure conditions, sleep in relaxed posture is difficult for cattle. Thus, exhibition of sleep-related lying postures might be an indicator of cattle safety and comfort. Using electrophysiological readings, Hunter et al [9] examined that cattle lying with its head tucked on flank posture would occasionally relate with rapid eye movement (REM) sleep, while both REM and non-REM sleep were recorded in cattle lying with its head extended on the ground. This suggests that different head positions during lying might have a different contribution to the sleep state of cattle. However, in previous studies, these sleep-related lying postures were only measured collectively. Measuring the tucked and extended head positions while lying separately could add a new aspect to the assessment of comfort during resting.

**Figure 1:**
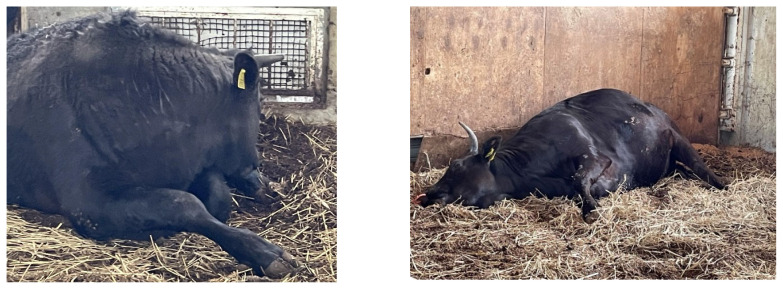
Examples of (Left) tucked sleep-related posture (TSP): cattle are recumbent with their necks turned backwards and their heads resting on the flank or on the ground; (Right) extended sleep-related posture (ESP): cattle are recumbent with their necks extended and with their heads on ground in front.

Therefore, this study aimed at determining how comfort during resting influences different sleep-related lying posture expressions. The cleanliness of bedding affects the comfort of cattle when resting [10–13]. We investigated the effects of bed cleanliness on the duration and frequency of sleep-related lying postures in cattle. Adopted from Hunter et al [9], we focused on two of the most visually distinguishable sleep-related lying postures (Figure 1): tucked postures (TSP), when cattle are recumbent with their necks turned backwards and their heads resting on the flank or ground, and extended postures (ESP), when cattle are recumbent with their necks extended and with their heads on the ground in front. We hypothesized that cattle would display sleep-related lying postures longer and more frequently following bed cleaning.

## MATERIALS AND METHODS

### Animals and management

The study was conducted at the Kawatabi Field Science Center in Tohoku University (Osaki, Japan) in April and May, 2021. The experimental procedure was approved by the Ethical Committee for Animal Experiments of Tohoku University (2020AgA-029-01).

Eight Japanese Black fattening cattle were subjected to this study (average body weight 575.0±45.4 kg, average age 19.7 ±1.7 months, four heifers and four steers). Two cattle were allocated into each pen (5×5 m2) based on their ages. Two adjacent pens were regarded as group (1, 2) and were allocated to two treatments: cleaning (CL) and control (CON). Each group consisted of a pen with two heifers and the other pen with two steers, respectively. The cattle were fed with straw and commercial concentrate (Shiratori Kouki 70; Meiji Feed, Tokyo, Japan) at 09:00 and 16:00 every day, and received water and salt *ad libitum*.

The cattle shed consisted of a concrete floor, concrete wall, and roof. Sawdust (3 m^3^/pen) was used as bedding material. The first experiment was conducted between April 5 and 16, 2021 (Period 1) and the second experiment was conducted between May 10 and 20, 2021 (Period 2). For all pens, the bedding material had not been replaced for one month before the study. To maintain similar hygiene levels in the different experimental periods, 0.5 m^3^ of compost was added to the surface of each pen once two weeks prior to the start of each period. The bedding material was replaced as a cleaning treatment in CL in the middle of each experimental period (April 12 and May 17 in period 1 and period 2, respectively), which was regarded as day 0. In CON, the bedding material was not replaced until the end of each experimental period. The study was replicated in experimental period 2 to secure sufficient sample size. The cattle in the CL were allocated to CON and vice versa in experimental period 2, following the same experimental procedure.

### Measurements

Behavioral measurements and samples for bedding cleanliness were taken on days −7, −5, 2, and 4 relative to the cleaning treatment (day 0) in period 1, and days −7, −4, 1, and 3 relative to day 0 in period 2. Blood samples for measuring plasma cortisol and serotonin concentrations were taken twice in each period on days −7 and 4 in period 1 and days −7 and 3 in period 2. The blood sampling was taken between 09:00 to 12:00 in the morning.

Sleep-related lying postures were recorded for 24 h using an infrared camera (WTW-EHR1152HJ2; Wireless Tsukamoto, Mie, Japan) and a digital video recorder (WTW-DEHP 104G-2TB; Wireless Tsukamoto, Japan). Two cameras were located at 4.0 m distance and 3.5 m above the pen. One camera could monitor two adjacent pens. The TSP and ESP were continuously observed and visually identified in the video. The duration and bout frequency of TSP and ESP per 24 h were measured in each cattle, and the mean bout length was calculated by dividing the duration with bout frequency. The total duration of sleep-related lying postures was calculated as the sum of the durations of TSP and ESP.

The ammonia (NH_3_) concentration in the air and moisture content of bedding materials in each pen were measured as indicators of bedding cleanliness. NH_3_ concentration that was 20 cm above the bedding surface in each pen was measured using a commercial gas collector (AP-20; Komyo Rikagaku Kogyo, Kawasaki, Japan). Bedding material was randomly collected from three locations in each pen and then pooled for a 100-g sample in each pen. The samples were dried for 48 h at 70°C, and the moisture content was calculated.

Blood samples (10 mL) were collected from the jugular vein of each animal via syringe transferred into vacuum tubes containing heparin, and centrifuged (4°C, 1,600 g, 15 min) for plasma collection. Plasma cortisol was measured using a commercial EIA kit (Detect X Cortisol EIA kit; ARBOR ASSAYS, Ann Arbor, MI, USA). The intra-assay coefficient was 25.8%. Plasma serotonin levels were measured using a commercial enzyme-linked immunosorbent assay (ELISA) kit (Serotonin ELISA kit; Enzo Life Sciences, East Farmingdale, NY, USA). The intra-assay coefficient was 4.6%.

### Statistical analysis

In each experimental period, all of the measurements were averaged per pen. In each experimental period, the mean of each behavioral and bedding cleanliness measurement taken on the days before and after day 0 were considered as “before cleaning” and “after cleaning”, respectively. Similarly, the results of blood samples taken before and after day 0 were considered as “before cleaning” and “after cleaning”, respectively. Measurements of bedding cleanliness, sleep-related lying postures, and plasma cortisol and serotonin concentrations were analyzed using linear mixed models with normal distribution (SPSS statistics 23, IBM). The experimental unit was the pen. To test the changes due to treatment before and after cleaning, the interaction between treatment (CL, CON) and measuring days (before cleaning, after cleaning) was included as a fixed effect. The effects of treatment, measurement days, group (1, 2), and experimental period (1, 2) were also included in the models as fixed effects. When there was interaction between the treatment and measuring days, pairwise comparison between CL and CON were performed before and after cleaning. The effects of pen (1 to 4) within a group and measuring day (days −7, −5, 2, and 4 in period 1 and days −7, −4, 1, and 3 in period 2) within an experimental period (1,2) were included as random effects. Statistical significance is defined when p-values are less than 0.05.

## RESULTS

The effects of bed cleaning on TSP and ESP are listed in Table 1. A total duration of sleep-related lying postures (sum of TSP and ESP) in CL was 104.4±29.4 min/d (mean±standard deviation) before bed cleaning and 121.5±22.8 min/d after bed cleaning. Those in CON were 106.6±20.3 min/d and 116.7±12.8 min/d, respectively. We observed significant differences associated with group (p<0.05); however, there was no interaction between treatment and measuring days in a total duration of sleep-related lying postures. There were significant interactions between treatment and measuring days in duration (p<0.05) and bout frequency (p<0.05) of TSP. The duration of TSP in CL was shorter than CON after bed cleaning (55.4±11.1 min/d vs 74.2±18.2 min/d; p<0.01). Similarly, the bout frequency of TSP in CL was less than CON after bed cleaning (9.6±1.3 times/d vs 11.8±1.8 times/d; p< 0.05). The experimental period affected the mean bout length of TSP (p<0.05); however, treatment and measuring days showed no interaction. There was a tendency of interaction between treatment and measuring days in daily duration of ESP (p = 0.052), and a significant interaction between treatment and measuring days was also found in bout frequency (p<0.01). The duration of ESP in CL was longer than CON after bed cleaning (66.2±26.4 min/d vs 42.4±12.7 min/d; p<0.05). Similarly, the bout frequency of ESP in CL was more than CON after bed cleaning (8.6±1.6 min/d vs 6.7±1.1 time/d; p<0.05). There was no interaction between treatment and measuring days in bout length of ESP. The duration (p<0.05), bout frequency (p<0.01), and bout length of ESP (p<0.05) varied significantly between groups.

Plasma cortisol concentration in CL before and after cleaning were 19.1±4.8 ng/mL and 16.6±2.5 ng/mL, and plasma cortisol concentration in CON before and after cleaning were 21.0±10.8 ng/mL and 19.0±6.8 ng/mL, respectively. Treatment and measuring days did not show any interactive effect on cortisol concentration. Plasma serotonin concentration in CL before and after cleaning were 897.2±415.6 ng/mL and 976.9±379.6 ng/mL, and serotonin concentrations in CON before and after cleaning were 971.2±315.8 ng/mL and 835.0±193.3 ng/mL, respectively. While the experimental period influenced serotonin concentration (p< 0.05), treatment and measuring days showed no influence.

The NH_3_ concentrations in the air and moisture of the bedding are listed in Table 2. There was an effect of measuring days (p<0.01) and there was interaction between treatment and measuring days (p<0.01) in NH_3_ concentration. Before cleaning, NH_3_ concentration in the air was higher in CL compared to CON (3.8±0.9 ppm vs 2.7±0.6 ppm; p<0.05). After cleaning, the NH_3_ concentration in CL was lower than that in CON (1.0±0.5 ppm vs 2.0±1.1 ppm; p<0.05). There were effects of treatment (p<0.01), measuring days (p<0.05), and their interactions (p<0.01) on bedding moisture. Moisture levels in bedding in the CL after bed cleaning was lower than that in CON (45.1%±4.8% vs 56.4%±3.3%; p<0.01).

## DISCUSSION

This study aimed to investigate the effects of bed cleaning on sleep-related lying postures in Japanese Black fattening cattle. The bedding moisture and NH_3_ concentrations, which are regarded as indicators of bed cleanliness, declined after bed cleaning treatment, indicating that bed cleanliness improved after cleaning.

Bed cleanliness did not significantly affect the total duration of sleep-related lying postures in our study. The present study was not in accordance with previous studies that reported an increase in the duration of sleep-related lying postures of cattle under enrichment treatment of housing conditions including more frequent bedding straw replacement [3] and, loose housing with deep bedding compared to tie stall [4]. The reason for such discrepancies remains unclear in this study; however, we speculate that it could be due to differences in experimental periods and treatment types. Previous studies were conducted for longer experimental periods ranging from 51 to 240 days. The present study also did not implement tie stalls, which might greatly restrict the head movement range of the cattle.

Unlike the total duration of sleep-related lying postures, changes in the duration and frequency of TSP and ESP after bed cleaning were observed in the present study, suggesting that changes in posture might be more sensitive in a relatively short experimental period. A longer duration and higher bout frequency of ESP, and a shorter duration and lower bout frequency of TSP in CL compared to CON were observed after bed cleaning. This suggests that the transition from TSP to ESP occurred in response to improved bed cleanliness. Since the bout length remained stable, a higher bout frequency of ESP was assumed to lead to a longer daily duration of ESP after bed cleaning. The result of an increase in ESP is consistent with Krohn and Munksgaard [4], who reported that cows had a longer duration (% of total lying time) for lying with their heads on the ground or flat on the side when they were on pasture compared to when they were in loose housing with deep bedding. Similar to the CON in our study, the lying postures of cows in Krohn and Munksgaard [4] may be affected by unclean conditions on deep bedding. Van Erp-van der Kooij et al [13] reported a higher proportion of cattle lying with their heads turned back in indoor cubicles than on pasture. This suggests that cattle might have no choice except displaying a compact lying posture like TSP when bed was polluted by feces. Cattle prefer dry and clean bedding, and they choose to lie on a clean bed over a wet or dirty bed in a loose house [14]. The present study suggests that providing comfortable resting places in indoor systems would allow cattle to display relaxed head positions while lying. The increase in ESP could also be due to an increase in the availability of resting spaces. Bulls spent more time in an outstretched body posture (lying on the side or on the belly with legs stretched out) as the indoor space allowance increased [15]. Similarly, in the present study, removing feces by bed cleaning provided more available resting space, and cattle might have been able to choose to display ESP, which is a more outstretched posture than TSP.

Changes in plasma cortisol and serotonin levels were not observed after cleaning. Blood cortisol level indicates the stress level of cattle under indoor housing [16], and serotonin concentration indicates positive changes in the welfare state of cattle [17]. Chen et al [10] compared the plasma cortisol levels of cattle under dry, muddy, and very muddy housing conditions and found no difference in cortisol levels under different cleanliness conditions. Although the present study showed that cattle show longer relaxed sleep-related lying postures in response to cleaning, they might not induce changes in their emotional states.

Hunter et al [9] revealed that cattle occasionally display TSP during REM sleep, and that ESP is partially related to REM and non-REM sleep. The electroencephalogram of cattle was not measured in our study; therefore, we cannot conclude whether the shift in lying postures was also due to changes in the sleep stages of cattle in cleaner environments. Future studies could explore the relationship between lying postures, sleep stage, and housing conditions by combining electroencephalogram and behavioral data of cattle.

The present study showed that cattle shifted their lying posture from tucked head positions to extended head positions in response to improved bed cleanliness, even though the total duration was unchanged. The extended head positions of sleep-related lying posture might be indicative of comfort around the resting state of cattle.

## Figures and Tables

**Table 1:** Duration, bout frequency, bout length of TSP and ESP, and total duration of sleep-related posture in cleaning (CL) and control (CON) treatment before and after cleaning (mean±standard deviation)

Items	Before cleaning		After cleaning		Model effects^1)^				
	
	CL	CON	CL	CON	T×D	T	D	G	P
Total sleep-related posture duration (min/d)	104.4±29.4	106.6±20.3	121.5±22.8	116.7±12.8	NS	NS	^†^	^*^	^†^
TSP									
Duration (min/d)	58.2±13.7	53.3±16.1	**55.4±11.1**	**74.2±18.2**	^*^	NS	NS	NS	NS
Bout frequency (time/d)	9.9±2.0	8.4±3.0	**9.6±1.3**	**11.8±1.8**	^*^	NS	NS	NS	NS
Bout length (min)	6.2±1.6	6.8±0.8	6.3±1.5	6.4±1.3	NS	NS	NS	NS	^*^
ESP									
Duration (min/d)	46.2±22.5	53.2±23.9	**66.2±26.4**	**42.4±12.7**	^†^	NS	NS	^*^	NS
Bout frequency (time/d)	5.7±2.1	7.2±2.5	**8.6±1.6**	**6.7±1.1**	^**^	NS	NS	^**^	NS
Bout length (min)	6.8±1.8	6.1±1.4	7.2±2.1	5.6±1.5	NS	^†^	NS	^*^	NS

TSP, tucked sleep-related posture; ESP, extended sleep-related posture.

1) T, treatments (CL, CON); D, measuring days (before cleaning, after cleaning); G, groups (1,2); P, experimental periods (1,2).

NS, not significant;

† p<0.1;

* p<0.05;

** p<0.01;

bold fonts indicate significant pairwise difference (p<0.05) between CL and CON.

**Table 2:** NH_3_ concentration in the air and bedding moisture in cleaning (CL) and control (CON) treatment groups before and after cleaning (mean±standard deviation)

Items	Before cleaning		After cleaning		Model effects^1)^				
		
	CL	CON	CL	CON	T×D	T	D	G	P
Ammonia in the air (ppm)	**3.8±0.9**	**2.7±0.6**	**1.0±0.5**	**2.0±1.1**	^**^	NS	^**^	NS	NS
Bedding moisture (%)	55.5±3.7	57.3±5.4	**45.1±4.8**	**56.4±3.3**	^**^	^**^	^*^	NS	NS

1) T, treatments (CL, CON); D, measuring days (before cleaning, after cleaning); G, groups (1,2); P, experimental periods (1,2).

NS, not significant;

* p<0.05;

** p<0.01;

bold fonts indicate significant pairwise difference (p<0.05) between CL and CON.
